# Pressure‐Induced Ultralow Critical Micelle Concentration of Surfactant for Encapsulating Dye

**DOI:** 10.1002/advs.202415151

**Published:** 2025-06-10

**Authors:** Qi Li, Xinze Liu, Peng Zhu, Meilin Guo, Guangxiong Hu, Jianbo Gao, Cailong Liu, Ying Shi

**Affiliations:** ^1^ Institute of Atomic and Molecular Physics Jilin University Changchun 130012 P. R. China; ^2^ Department of Chemistry Brock University St. Catharines ON L2S 3A1 Canada; ^3^ Key Laboratory of Quantum Materials Under Extreme Conditions in Shandong Province, School of Physics Science & Information Technology Liaocheng University Liaocheng 252059 P. R. China

**Keywords:** cetyl trimethyl ammonium bromide, coumarin, pressure, ultralow critical micelle concentration

## Abstract

Achieving the ultralow critical micelle concentration (CMC) of the surfactant encapsulating the dye has spawned a vital pathway to boost the efficiency and stability of dye‐sensitized solar cells (DSSCs). The attainment of ultralow CMC while sustaining the constant micelle presents a significant challenge. Here, by an ingenious pressurizing strategy, the ultralow CMC is obtained for the immobilized micelle. The ultralow CMC of cetyl trimethyl ammonium bromide (CTAB) is performed under pressure by the detection of encapsulated coumarin 35 (C35) attractively. At 0.80 GPa, the CMC of CTAB is decreased ≈111‐fold to 8.3 ± 0.1 µm compared to 917.2 ± 8.7 µm at 0.10 MPa. The ultralow CMC under pressure is probed relying on the quenched fluorescence background of C35. Femtosecond transient absorption spectra confer a clear picture that the pressure‐induced fluorescence quenching is ascribed to the reinforced competition for the twisted intramolecular charge transfer process of the C35. This study provides a robust strategy for achieving ultralow CMC, laying the groundwork for the efficient application of DSSCs.

## Graphical abstract

Ultralow critical micelle concentration (CMC) is successfully achieved by pressure. Compared to 917.2 ± 8.7 µm for cetyl trimethyl ammonium bromide at 0.10 MPa, the ultralow CMC at 0.80 GPa is a ≈111‐fold reduction for 8.3 ± 0.1 µm. The detection of ultralow CMC under pressure is owed to the pressure‐induced fluorescence quenching of the coumarin 35 probe.

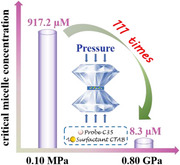

## 1 Introduction

Dye‐sensitized solar cells (DSSCs) have been at the forefront of scientific exploration as a low‐cost and high‐efficiency photovoltaic technology.^[^ ^1^, ^2^, ^3^, ^4^, ^5^, ^6^ ^]^ Coumarin dyes and their derivatives with broad absorption spectra and high molar extinction coefficients are powerful candidates for sensitizers in DSSCs.^[^ ^7^, ^8^, ^9^, ^10^, ^11^, ^12^ ^]^ Concerningly, the photoelectric conversion efficiency is severely hindered by the aggregation of photoactive dye.^[^ ^13^, ^14^, ^15^, ^16^ ^]^ To address this issue, the micellar‐wrapped dye strategy has emerged as a prominent solution due to its remarkable capabilities in self‐assembly and spatial confinement for dyes.^[^ ^17^, ^18^, ^19^, ^20^, ^21^, ^22^ ^]^ The incorporation of ionic surfactants has been demonstrated to substantially improve device performance through synergistic enhancement of the photovoltaic parameters.^[^ ^19^, ^23^ ^]^ This enhancement is exemplified by the introduction of N,N,N‐trimethyl‐3‐(perfluorooctylsulfonamido)propan‐1‐aminium iodide, which elevates power conversion efficiency from 2.51% to 3.96% through surfactant mediation.^[^ ^23^ ^]^ Remarkably, gained by micelle cetyl trimethyl ammonium bromide (CTAB) packed dyes, the efficiency of the cell is increased by 50‐fold and the storage capacity is extended to 4–5 days in contrast to without it.^[^ ^19^ ^]^ These collective advances position micellar encapsulation as a versatile platform for developing high‐performance and aggregation‐resistant dye systems in photovoltaic applications.

The critical micelle concentration (CMC) is a fundamental and pivotal indicator in dye‐enveloped micellar systems.^[^ ^24^, ^25^ ^]^ The micelle is formed as the surfactant exceeds this specific threshold.^[^ ^25^ ^]^ It is generally recognized that adopting surfactants around CMC is propitious for achieving maximum electrical output.^[^ ^26^, ^27^ ^]^ Surprisingly, the micelles serving as carriers with lower CMC contribute to both remarkable efficiency and superior stability.^[^ ^28^, ^29^, ^30^, ^31^, ^32^ ^]^ Current methodologies for reducing CMC encompass two distinct approaches, i.e., modification of micellar assemblies and preservation of native micellar configurations.^[^ ^31^, ^32^, ^33^, ^34^, ^35^, ^36^, ^37^ ^]^ In particular, the fixed micelle strategy merits heightened attention, as exemplified by CTAB surfactant, enabling simultaneous CMC optimization and maintenance of superior charge transport efficiency.^[^ ^18^ ^]^ For constant CTAB surfactants, there are three main strategies to achieve the variations of CMC, namely, temperature, electrolyte, and additives.^[^ ^34^, ^35^, ^36^, ^37^ ^]^ For the temperature strategy, the CMC of CTAB is lowest at ambient temperature and displays a gradual increment with increasing temperature.^[^ ^34^, ^35^ ^]^ The growth in electrolyte concentration results in a decrease in the CMC of CTAB from 990.0 to 120.0 µm.^[^ ^36^ ^]^ The ratio of silicone oil additives also affects the CMC of CTAB. Specifically, the CMC of CTAB decreases from 2160.0 µm at oil content at 45.7% to 1180 µm at 12.3%.^[^ ^37^ ^]^ However, the impasse of current techniques typically falls short in decreasing CMC. Obtaining ultralow CMC while holding micellar conformation remains an urgent challenge.

Herein, we present a pressurized method for accessing ultralow CMC in the fixed CTAB surfactant system. The microenvironment‐sensitive coumarin 35 (C35) dye serves as a dexterous probe for evaluating the CMC of CTAB. The CMC of CTAB at atmospheric pressure detected by C35 is ≈917.2 µm. Under pressure, the fluorescence of C35 is dramatically quenched, avoiding the obstruction of a strong fluorescence background to exploring ultralow CMC. Wondrously, the CMC of CTAB probed by C35 at 0.80 GPa is reduced by 111‐fold to ≈8.3 µm compared to 0.10 MPa. The availability of C35 under pressure for ultralow CMC is greatly fostered by the competition of the twisted intramolecular charge transfer (TICT) process. It is revealed by femtosecond transient absorption (fs‐TA) spectra as well as Raman spectra. This work could substantially resolve the issue of achieving ultralow CMC without micellar modification, providing a feasible strategy for developing DSSCs.

## 2 Results and Discussion

### 2.1 CMC Detection of CTAB at Atmospheric Pressure by C35

**Figure 1:**
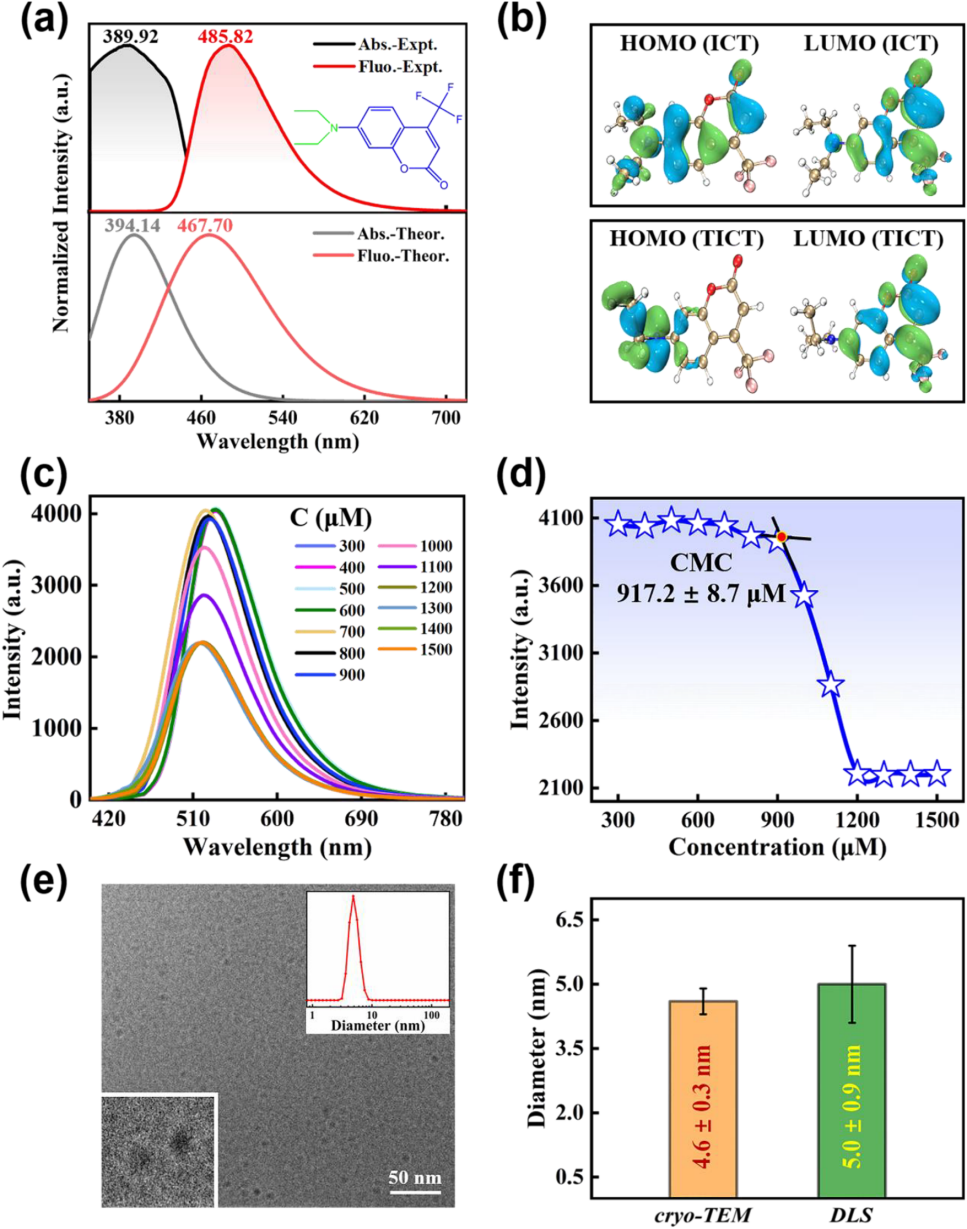
a) Experimentally and theoretically normalized absorption and emission spectra of C35 in THF at atmospheric pressure. The inset portrays the molecular structure of C35. b) Modeled frontier molecular orbitals of the ICT state and the TICT state. c) Fluorescence spectra with different [CTAB] (µm) in aqueous solution using the C35 probe. d) Plot of fluorescence peak intensity vs. different concentrations of CTAB. CMC was demonstrated by mean value ± standard deviation (three parallel experiments by fluorimetry). e) Cryo‐TEM image of 1500 µm CTAB; scale bars: 50 nm. The inset is the size distribution of 1500 µm CTAB measured by DLS. f) Hydrodynamic diameters and corresponding errors of CTAB measured by cryo‐TEM and DLS.

Recognizing the excited‐state structural behavior of photosensitizers is a prerequisite for the actual deployment. The experimentally measured and theoretically modeled absorption and fluorescence spectra of C35 at atmospheric pressure are shown in Figure 1a. The measured absorption peak is situated at 389.92 nm. In conjunction with the simulated absorption peak of 394.14 nm in the S_1_ state, the sample is prepared to the S_1_ state under excitation at 400 nm. The C35 dissolved in THF exhibits a single fluorescence peak at 485.82 nm over the concentration range from 0.01 to 5 mm (Figure S1, Supporting Information). It indicates that the C35 in THF is stored as a monomeric form in our study. Sequentially, the root of the fluorescence is identified. In **Figure** **1**a, the fluorescence calculated from the ICT state is 467.70 nm, which coincides with the experimentally recorded fluorescence peak at 485.82 nm. Therefore, the single fluorescence peak is attributed to the emission of the ICT state. The TICT state with lower energy than the ICT state is attained in the structural simulations in Figure S2 (Supporting Information). The charge distribution of the TICT state in Figure 1b exhibits an obvious differentiation between the donor and acceptor with rotation of the diethylamino group. The overlap degree of charge between the highest occupied molecular orbital (HOMO) and lowest unoccupied molecular orbital (LUMO) is sufficiently small that the TICT state is non‐fluorescence.^[^ ^38^, ^39^, ^40^, ^41^ ^]^ Thus, the fluorescence of the C35 is emitted in the ICT state, while the TICT state is the dark state.

The fluorescence spectra of C35 with different concentrations of CTAB at atmospheric pressure are depicted in Figure 1c. The fluorescence intensity of the C35 probe is localized ≈4050.00 a.u. with CTAB concentrations ranging from 300 to 900 µm. Thereafter, the fluorescence is weakened at CTAB concentrations up to 1000 µm and levels off at 1200 µm. The variation in fluorescence intensity is scrutinized in Figure 1d. The tangent through the sigmoid curve demonstrates that the CMC of CTAB is 917.2 ± 8.7 µm probed by C35, consistent with those reported in the literature.^[^ ^42^, ^43^ ^]^ It should be noted that the fluorescence intensity is contracted above CMC, confirming the hydrophobic effect of the C35 probe, i.e., aggregation‐caused quenching as evinced in Figure S4 (Supporting Information). As the volume of water gradually increases, the fluorescence is uninterruptedly weak. In this case, the aggregation of C35 derives from the increase in the localized concentration of C35 during the conversion of the CTAB surfactant from the amphiphilic molecules to the confined micelle. The cryogenic transmission electron microscopy (cryo‐TEM) is employed as a reliable tool to characterize the micelles.^[^ ^44^ ^]^ The cryo‐TEM image of CTAB concentration in 1500 µm is acquired in Figure 1e. As is visible, the spherical CTAB micelles are formed upon crossing the CMC of CTAB. The average diameter of the micelles is 4.6 ± 0.3 nm, which is statistically derived from the cryo‐TEM image by the Nano Measurer software. Based on the inset of dynamic light scattering (DLS) in Figure 1e, the hydrodynamic diameter of the CTAB micelles is 5.0 ± 0.9 nm. It is in line with the results obtained from the cryo‐TEM image. The hydrodynamic diameters and corresponding errors of the CTAB measured by cryo‐TEM and DLS are visualized in Figure 1f. Therefore, a convincing CMC of CTAB at atmospheric pressure is detected by the C35 probe.

### 2.2 Pressure‐Induced Ultralow CMC of CTAB and Probed by C35

**Figure 2:**
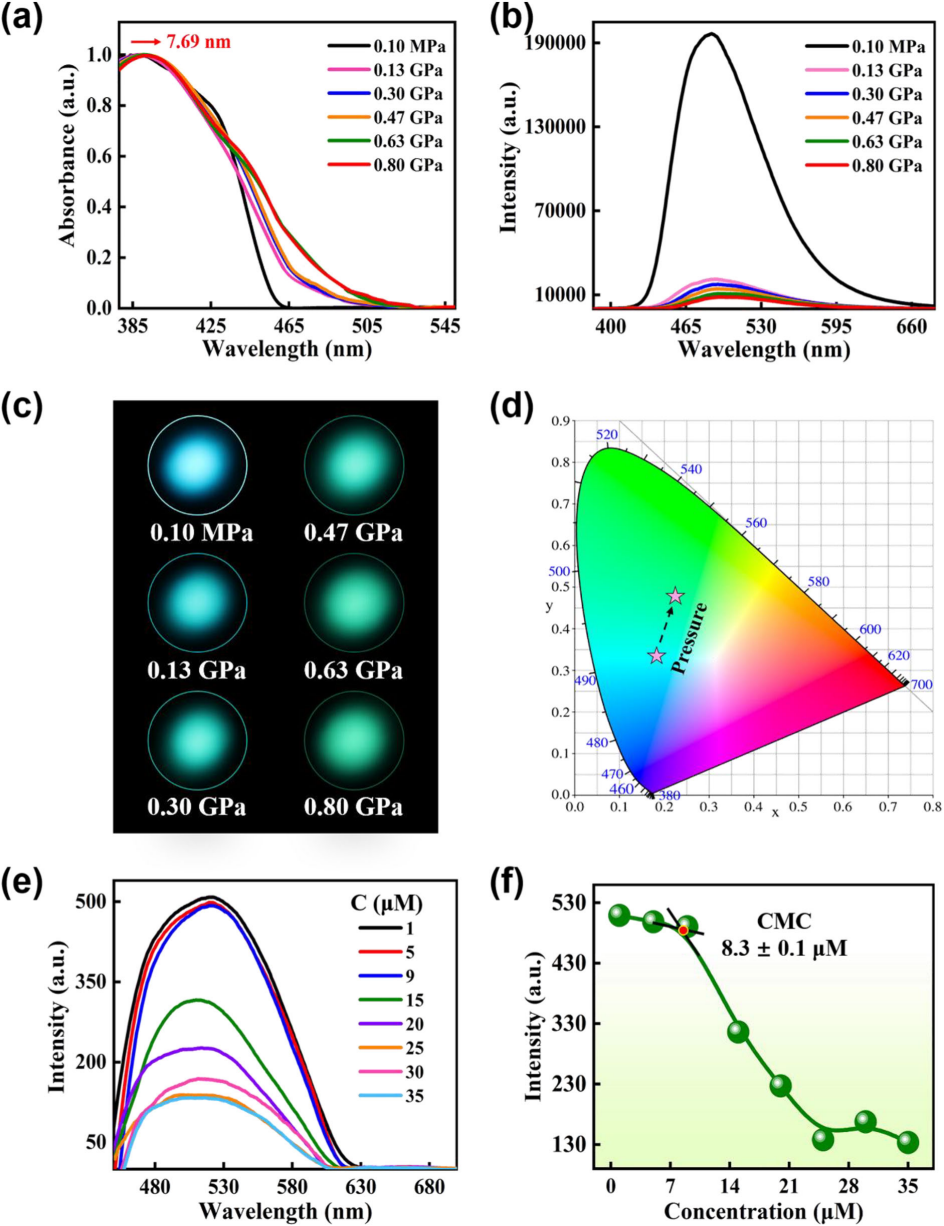
a) Normalized steady‐state absorption spectra of the C35 in the range of 0.10 MPa to 0.80 GPa. b) Steady‐state fluorescence spectra ranging from 0.10 MPa to 0.80 GPa. c) Fluorescence imaging of C35 under different pressures. d) Pressure‐dependent chromaticity coordinates for the emission of the C35. e) Fluorescence spectra for different concentrations of CTAB probed by C35 at a pressure fixed at 0.80 GPa. f) Plot of fluorescence intensity vs. [CTAB] (µm) at 0.80 GPa. CMC expressed as mean value ± standard deviation (three parallel experiments by fluorimetry).

The measurement of ultralow CMC is limited by the high fluorescence background of the coumarin probe.^[^ ^43^ ^]^ Primitively, the effect of pressure on the probe C35 is explored. The absorption spectra under pressure are shown in **Figure** **2**a. The redshift of the absorption peak by 7.69 nm is observed as the pressure is increased from 0.10 MPa to 0.80 GPa. It is attributed to the reduction of the energy gap between the S_0_ state and the ICT state under pressure. The fluorescence spectra of the individual C35 probe under pressure are carried out in Figure 2b. As the pressure grows, a significant fluorescence quenching is discerned, accompanied by a redshift of the peak position. The redshift of the fluorescence peak from 485.82 nm at 0.10 MPa to 502.77 nm at 0.80 GPa is equally assigned to the decrease in the energy gap between the S_0_ and ICT states under pressure. Exhaustively, the change in fluorescence intensity is discussed. In Figure 2b, the fluorescence intensity of C35 at 0.10 MPa is recognized as 196479.92 a.u. and is sharply reduced at pressure increases to 0.13 GPa. The fluorescence intensity is narrowed consistently to 8227.22 a.u. at 0.80 GPa, ≈24‐fold reduction compared to 0.10 MPa. The fluorescence imaging of Figure 2c displays the changing intensity from a visual perspective. As the pressure rises from 0.10 MPa to 0.80 GPa, the fluorescence intensity gradually fades. The redshift of the fluorescence peak under pressure gives rise to a modification in the color of the C35 probe. The color of the fluorescence is switched from cyan to greenish, as evidenced by the conversion of the CIE chromaticity diagram from (0.18, 0.34) to (0.22, 0.47) in Figure 2d. In this regard, the significant fluorescence quenching of C35 under pressure provides sufficient preparation for the detection of ultralow CMC.

Further, the contribution of pressure to the CMC of CTAB is treated by the quenched fluorescence of the C35 probe. As water is added during the CMC measurement, the phase transition of water under pressure of 0.90 GPa needs to be ascertained in advance.^[^ ^45^, ^46^, ^47^ ^]^ Therefore, the pressure is fixed at 0.80 GPa as the sample concentration is altered, corresponding to the maximum pressure of the liquid‐phase water in our study.^[^ ^47^ ^]^ Figure 2e delineates the fluorescence spectra of low concentrations of CTAB probed by C35. The fluorescence intensity is maintained at 520.00 a.u. when the CTAB concentration ranges from 1 to 9 µm. The fluorescence intensity is sustainably attenuated starting from a CTAB concentration of 15 µm and stabilized after 25 µm. Similarly to the fluorescence quenching during CMC probing at atmospheric pressure, the fluorescence quenching of CMC detection at 0.80 GPa is also attributed to the localized aggregation of C35 after the formation of nanoscale confined micelles. The detailed fluorescence intensity variation is extracted in Figure 2f. Unexpectedly, the CMC of CTAB at 0.80 GPa is 8.3 ± 0.1 µm, almost 111‐fold decrease compared to 0.10 MPa. It means that pressure is a valuable method for obtaining ultralow CMC in the surfactant CTAB‐encapsulated coumarin system. Thus, the ultralow CMC of surfactant CTAB is achieved by pressurization.

### 2.3 Raman Spectra at Different Pressures

**Figure 3:**
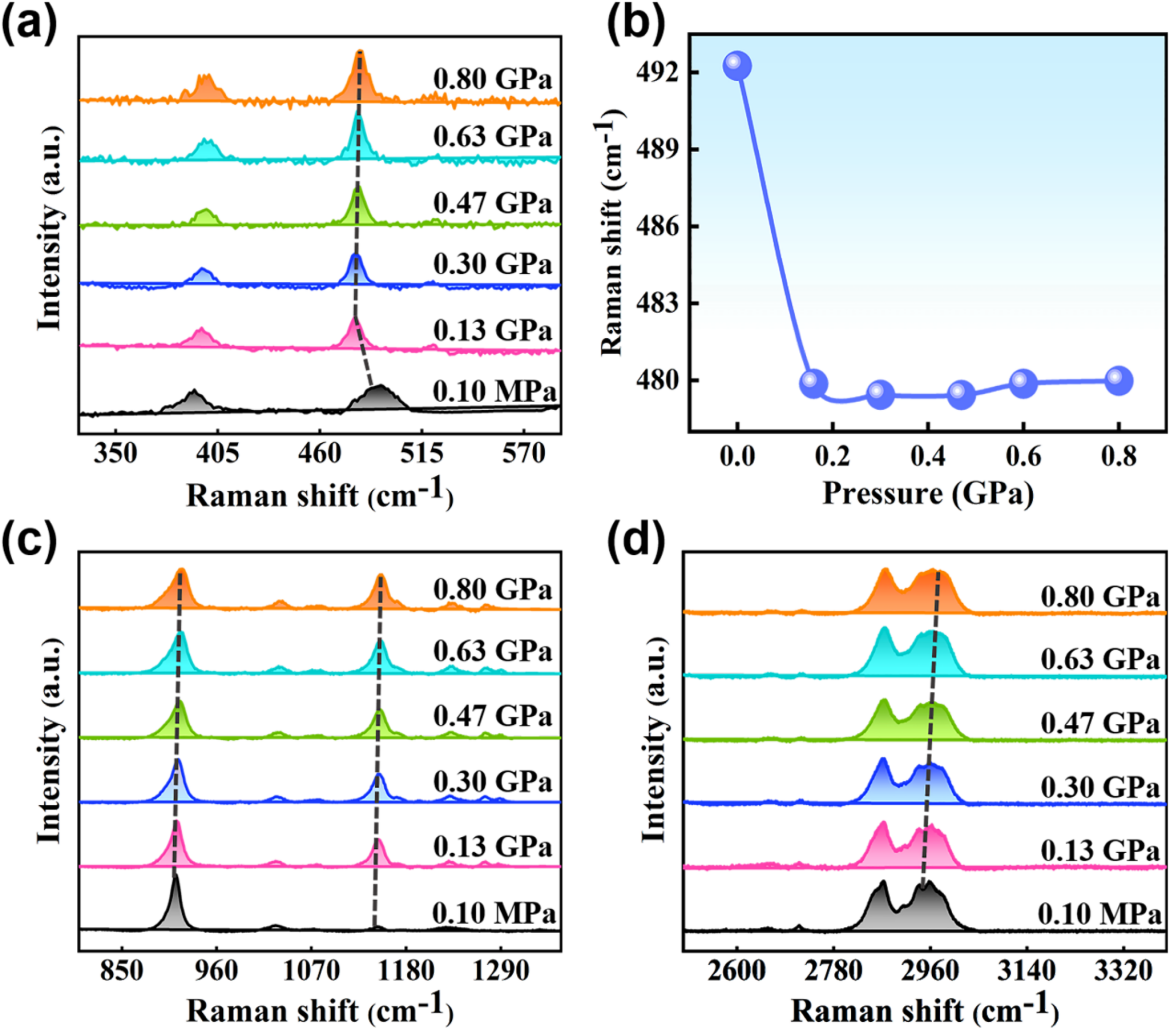
In situ high‐pressure Raman spectra characterizing the vibrations of intramolecular groups: a) 330–650 cm^−1^. b) Raman shift of the C─N bond as a function of pressure. c) 800–1360 cm^−1^. d) 2500–3400 cm^−1^.

The change of molecular conformation under pressure is tracked by Raman spectra. The Raman spectra at atmospheric and high pressures are displayed in **Figure** **3**. In virtue of the Raman spectral simulations as illustrated in Figure S5 and Table S1 (Supporting Information), the Raman shifts in the experiment are reasonably imputed. In detail, the Raman shift of 492.26 cm^−1^ at 0.10 MPa in Figure 3a is in agreement with the calculated Raman shift of 491.94 cm^−1^, which is attributed to the C─N bending vibrations of the diethylamino group. The Raman peak of the C─N bond is shifted to a lower frequency by pressurizing from 0.10 MPa to 0.13 GPa, demonstrating the enhancement of the bending vibrations of the C─N bond under pressure. The molecular deformation is produced under pressure.^[^ ^48^ ^]^ Continuously, the Raman shift is almost maintained with increasing pressure. The above description is meticulously portrayed in Figure 3b. In addition, the Raman shifts associated with the vibration of the C─H bond and the stretching of the C─N bond in Figure 3c,d are commonly blue‐shifted. The shortening of interatomic distances under pressure results in the Raman peaks shifting to a higher frequency.^[^ ^49^ ^]^ Also, there are no new peaks appearing nor old peaks disappearing in the Raman spectra under pressure. It means that the pressure evokes a conformational change corresponding to immobilized C35 rather than a phase transition.^[^ ^50^ ^]^ The exploration of Raman spectra proves that the bending vibrations of the distorted group induced by pressure are enhanced.

### 2.4 In Situ High‐Pressure Ultrafast Dynamics of C35

**Figure 4:**
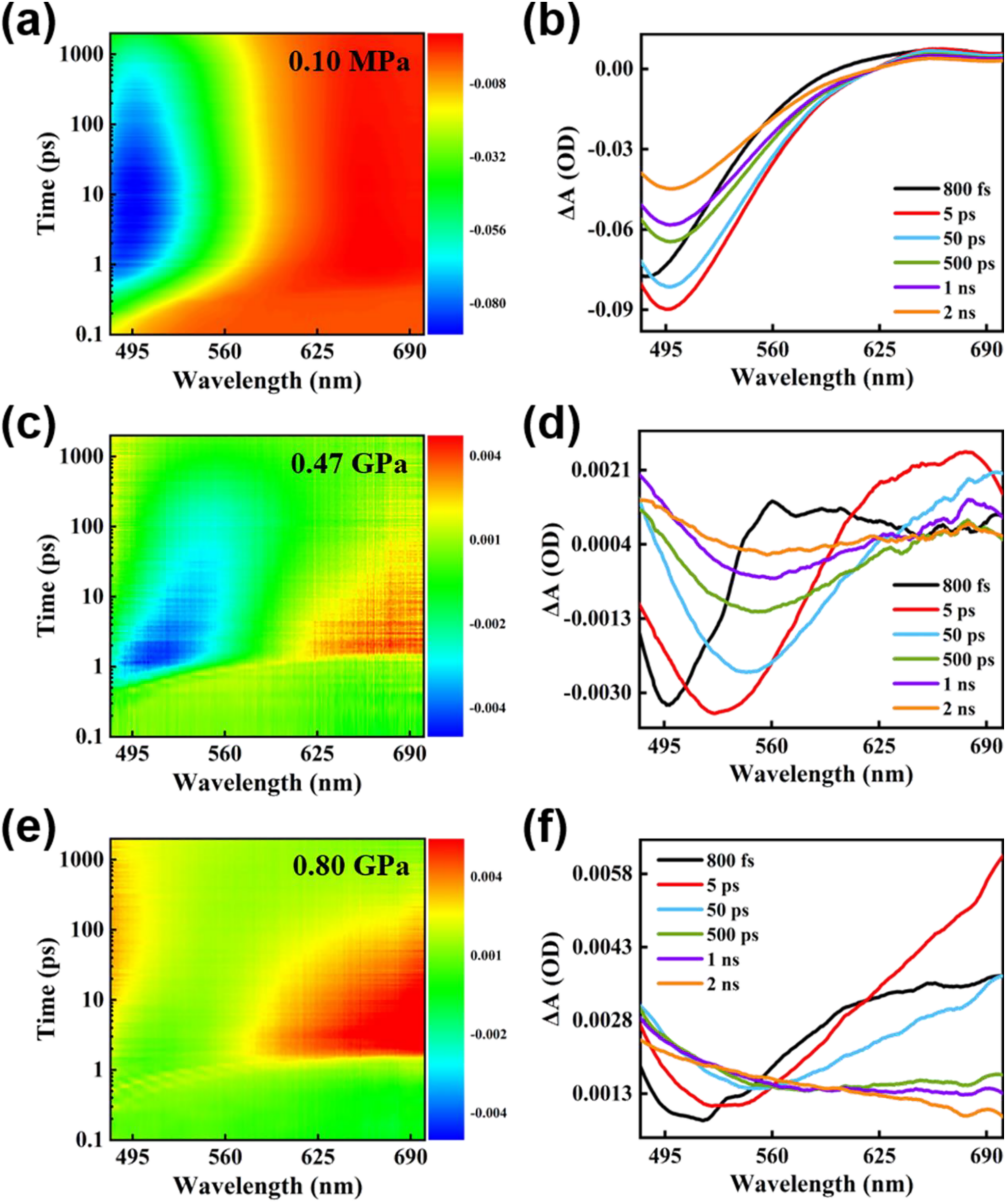
TA spectra and representative TA spectra at different time delays of C35 in THF at a,b) 0.10 MPa, c,d) 0.47 GPa, and e,f) 0.80 GPa.

The ultrafast dynamics under pressure are conducive to elucidating the pressure‐induced fluorescence quenching of the C35 probe. The fs‐TA spectra of C35 are exhausted in **Figure** **4** with the application of pressure. According to the fs‐TA at atmospheric pressure presented in Figure 4a, a significant stimulated emission (SE) is rendered at 484.00 nm, coinciding with the steady‐state fluorescence peak of 485.82 nm at 0.10 MPa.^[^ ^51^ ^]^ The SE signal is attainable for over 2 ns and gradually red‐shifted with time, which is proved by the TA signals with different time delays in Figure 4b. The effect of pressure on the C35 probe is explicitly discussed by the in situ high‐pressure fs‐TA spectra depicted in Figure 4c,e. It can be clearly seen that the fs‐TA spectra of the C35 under pressure are notably different compared to those at atmospheric pressure. Gradually pressurized from 0.10 MPa to 0.47 GPa, the weakened SE peak is observed, accompanied by a visible redshift, as captured in Figure 4d. At 0.80 GPa, the fs‐TA spectra at different time delays in Figure 4f demonstrate that the signal is converted to a positive absorbance signal of the excited state. The amplitude of the SE signal under pressure is weakened asymptotically.

**Figure 5:**
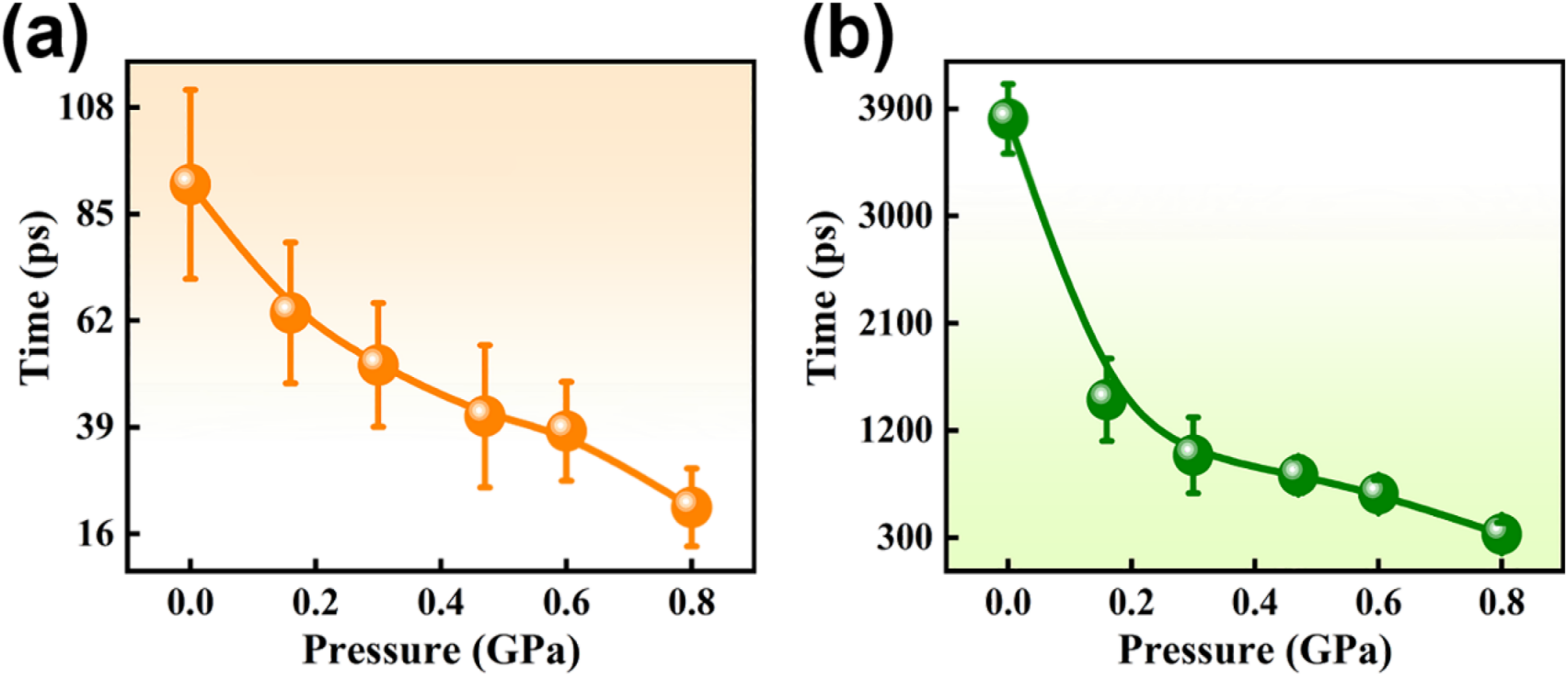
a) Lifetime constant of the conversion from the ICT state to the TICT state, and b) Fluorescence lifetime from the ICT state at different pressures. Error bars are labeled.

To explore the relaxation channels, bi‐exponential decay functions are convolved with the instrument response function to fit the fs‐TA spectra globally. The fitted lifetimes of the two ultrafast processes under pressure are gleaned in **Figure** **5**a,b. At 0.10 MPa, an initial delay of 91.43 ps and a long delay of 3816.00 ps are included. Drawing on the charge distribution and structural simulation in Figure 1b and Figure S2 (Supporting Information), the ICT state exists in the excited C35, and the consequent fluorescence is emitted. With the twisting of the diethylamino group, the TICT process is experienced. Thus, the process of a few tens of picoseconds (τ_1_) is classified as converting from the ICT state to the TICT state. The slower nanosecond‐scale (τ_2_) process is assigned to the fluorescence lifetime emitted from the ICT state. As the pressure grows from 0.10 MPa to 0.80 GPa, the transition lifetime from the ICT state to the TICT state is shortened from 91.43 to 21.76 ps. The energy barrier can be described by the Arrhenius equation effectively from the ICT state to the TICT state.^[^ ^52^, ^53^ ^]^

(1) $$k=A\exp\left(\frac{-E_{a}}{\mathrm{RT}}\right)$$

where k represents the rate of the ultrafast process, A is the prefactor, E_a_ denotes the activation energy, which can be obtained from the energy gap between the ICT state and the transition state, T is the temperature, and R is the universal gas constant. Thus, the energy barrier is decreased as the pressure increases based on the fitting lifetime from ICT to TICT transition shown in Figure 5a and the Arrhenius equation. Turning to the fluorescence lifetime, it is noticeably accelerated from 3816.00 ps at 0.10 MPa to 330.30 ps at harvesting the ultralow CMC of 0.80 GPa. These imply that more particles under pressure migrate from the ICT state to the non‐fluorescent TICT state upon excitation of C35. The gradual quenching of fluorescence under pressure is unfolded.

**Figure 6:**
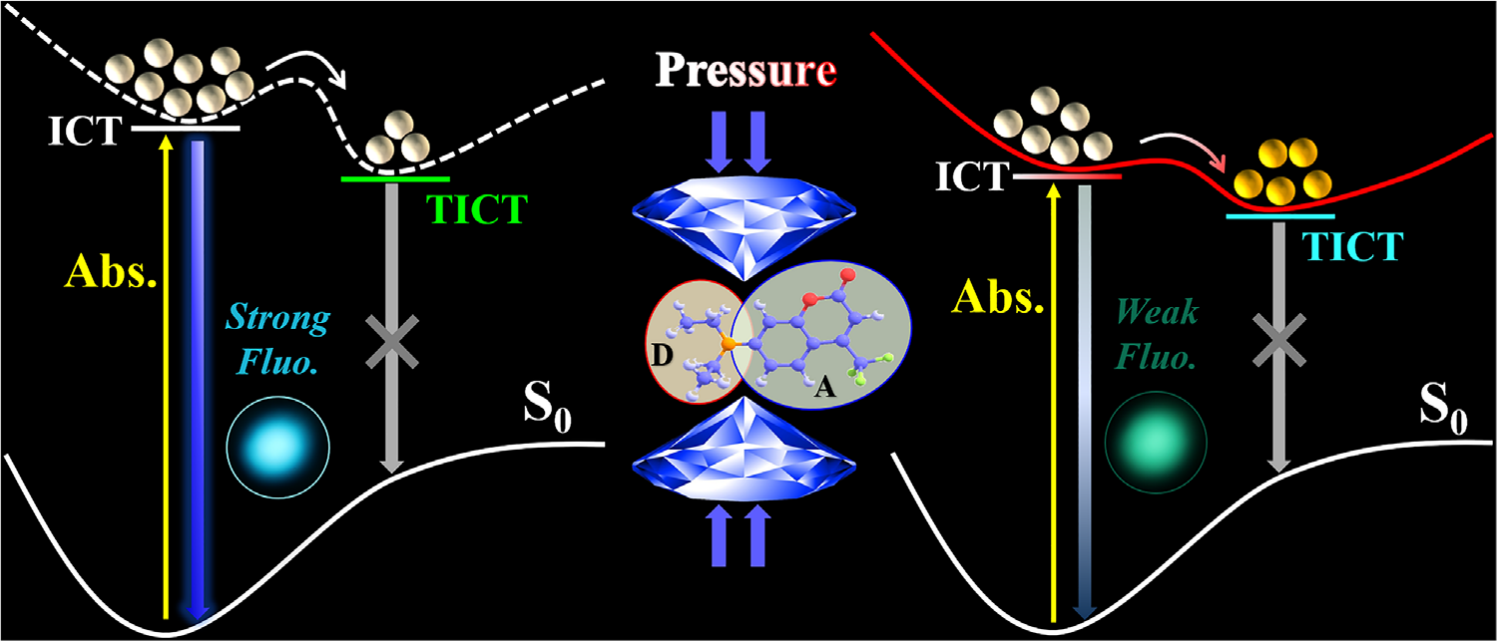
Schematic of the ultrafast dynamics of C35 at atmospheric pressure and under compression.

Building on the pressure‐induced lifetimes by fs‐TA spectra, the schematic representation for the evolution in the ultrafast process of C35 under pressure is mapped in **Figure** **6**. At atmospheric pressure, C35 is excited to the ICT state and emitted strong fluorescence. Subsequently, the non‐fluorescent TICT state is formed with lower energy than the ICT state. Pressure is introduced subtly to counteract the strong fluorescence background of C35. Under pressure, the energy gaps exhibit a universal reduction between both the ICT state and S_0_ state, as well as between the TICT state and S_0_ state. Compared to the lowered energy levels of the TICT state, the ICT state exhibits a pronounced energy reduction. Consequently, the energy gap between the ICT and TICT states under pressure becomes smaller than that at atmospheric pressure. It results in a progressive acceleration of the ICT‐to‐TICT transition with smaller energy barriers under pressure compared to atmospheric pressure. More particles are transferred to the non‐fluorescent TICT state while a minority of particles are emitted fluorescence in the ICT state. It is gratifying that it triggers the fluorescence quenching under pressure and enables the successful detection of pressure‐induced ultralow CMC.

## 3 Conclusion

In summary, the ultralow CMC has been successfully achieved in the promising CTAB system by pressure. Concretely, the CMC of CTAB probed by the ICT‐state fluorescence of C35 dye is ≈917.2 µm at atmospheric pressure. At the pressure of 0.80 GPa, the ultralow CMC of ≈8.3 µm is determined from the dark fluorescence background of the C35. The fluorescence quenching of the C35 probe that enables pressure‐induced ultralow CMC detection is elucidated by Raman spectra and fs‐TA spectra. The Raman spectra under pressure indicated that the bending vibrations of the rotated group are reinforced. As unveiled by the fs‐TA spectra of the C35 probe, the conversion from the ICT state to the TICT state with lower energy is accelerated. It provokes fluorescence quenching under pressure and enables the detection of pressure‐induced ultralow CMC. The encouraging result of ultralow CMC establishes the guideline for optimizing the performance of DSSCs.

## Conflict of Interest

The authors declare no conflict of interest.

## Supporting information



Supporting Information

## Data Availability

The data that support the findings of this study are available from the corresponding author upon reasonable request.
