# Supplementary material for: Pressure‐Induced Ultralow Critical Micelle Concentration of Surfactant for Encapsulating Dye
Source: Adv Sci (Weinh). 2025 Jun 10;12(33):e15151. doi: 10.1002/advs.202415151 (PMC12412479; doi:10.1002/advs.202415151)
Supplement: Supplementary file 1 — Supporting Information [file ADVS-12-e15151-s001.docx]

Supporting Information

**Pressure-Induced Ultralow Critical Micelle Concentration of Surfactant for Encapsulating Dye**

*Qi Li, Xinze Liu, Peng Zhu, Meilin Guo, Guangxiong Hu, Jianbo Gao, Cailong Liu,* and Ying Shi**

**Experimental Section and Theoretical Calculation**

***Materials:*** The C35 was purchased from Exciton Chemical Company without further purification. The THF with spectroscopy grade was obtained from Macklin. The CTAB was supplied by Sigma-Aldrich.

***Sample Preparation*:** The C35 was diluted in THF at a concentration of 5 mM. The CTAB was solubilized in distilled water. The initial concentrations of CTAB aqueous solution were configured to 1500 and 400 µM for detecting the CMC at atmospheric and high pressures, respectively. The C35 stock solution was microtitrated (40 µL) in a 5 ml volumetric flask and the THF was volatilized fully at room temperature overnight on a shaker.^[1,2]^ The CTAB aqueous solution was mixed in different volumes. The distilled water was added to the mark to produce a series of samples for fluorescence determination. The characterization of Raman spectra demonstrated that trace amounts of THF remained in the CTAB samples, as shown in Figure S3. The presence of a small quantity of THF in water did not affect the micellar system properties.^[3,4]^

***Pressure Generating Apparatus*:** The hydrostatic pressure is produced by a diamond anvil cell.^[5,6]^ The sample chamber was constructed with a T301 steel sheet with a 500 µm diameter and a 300 µm thickness. The ruby was applied for pressure calibration by the shift of the fluorescence peak under pressure.^[7]^ The THF and distilled water were used as pressure-transmitting medium for the isolated C35 dye and C35-CTAB mixture, respectively.

***Characterization*:** The deuterium lamp was determined by the absorption spectra. The C35 and C35-CTAB samples were excited by a 405 nm laser to obtain fluorescence spectra. The cryo-TEM image and DLS of CTAB were monitored by FEI Talos F200C and Malvern Zetasizer Nano ZS90, respectively. The Raman spectra were mapped by a Renishaw inVia microconfocal Raman spectrometer with a 532 nm semiconductor laser. The experimental conditions for fs-TA spectra were made by the ultrafast laser (Spectra-Physics, Solstice) with an 800 nm center wavelength, a 35 fs pulse duration, a 7 mJ pulse energy, and a 1 kHz repetition frequency.^[8,9]^ The 800 nm fundamental beam was divided into two beams in a 9:1 ratio. The pump pulse was multiplied to 400 nm by a β-BaB_2_O_4_ crystal to excite the sample. A super-continuum white probe pulse in the area of 450 nm to 800 nm was produced by the sapphire. The pump and probe pulses were combined at a small angle (θ≤5˚) in the sample cell. The detection was performed using a spectrometer (HELIOS). The acquired fs-TA spectra were globally fitted adopting Surface Xplorer software.

***Theoretical Calculation*:** The stable configurations of the ground and excited states were derived by the Gaussian 16 procedure.^[10]^ The B3LYP functional and TZVP basis sets were chosen to simulate electronic spectra that are in good agreement with experiments.^[11,12]^ The frontier molecular orbitals were generated by the Multiwfn program.^[13]^ The impact of THF solvent at atmospheric pressure was considered by the integral equation formalism polarizable continuum model.^[14]^

***Statistical Analysis***: The absorption and fluorescence spectra of C35 at atmospheric pressure were normalized. The CMC was expressed as the mean value ± standard deviation of three parallel experiments by fluorimetry. The size and corresponding error of the CTAB micelles in the cryo-TEM image were gained statistically by the Nano Measurer software (Fudan University, China). The figures were plotted through Origin software.

**
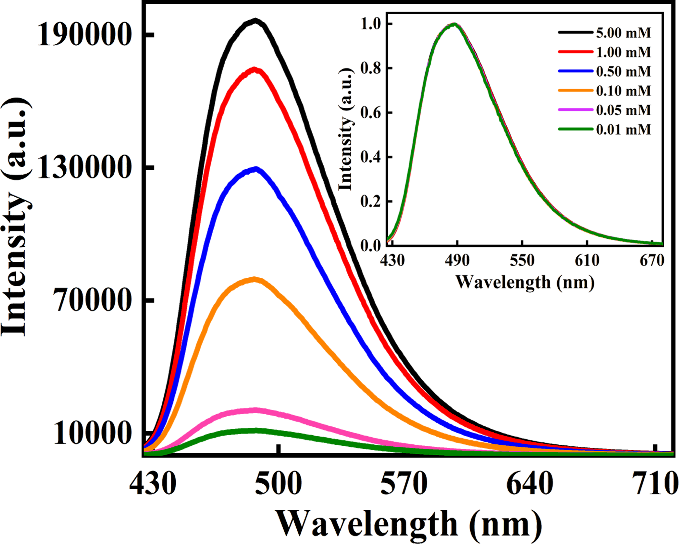
**

**Figure S1.** Fluorescence spectra of C35 at different concentrations. The inset represents the normalized fluorescence spectra.

**
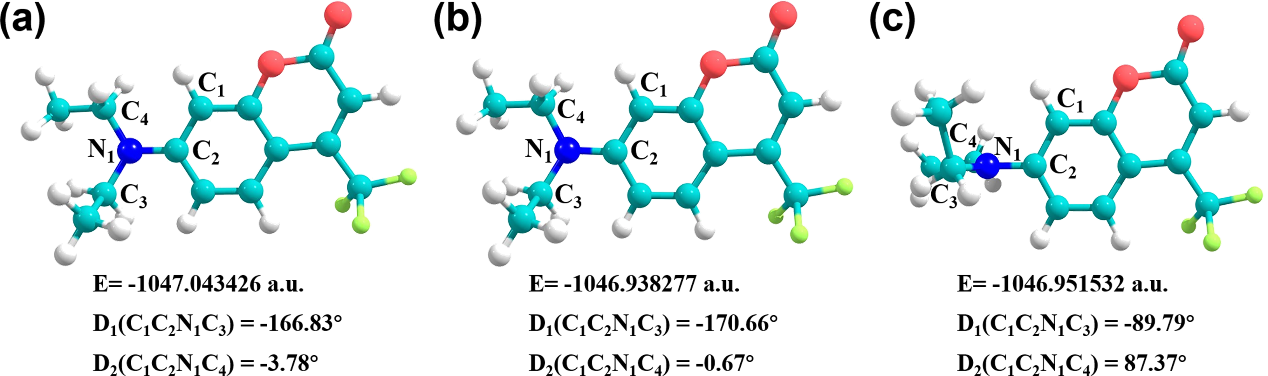
**

**Figure S2.** Modeled structures of the a) S_0_ state, b) S_1_ state, and c) TICT state of C35.

**
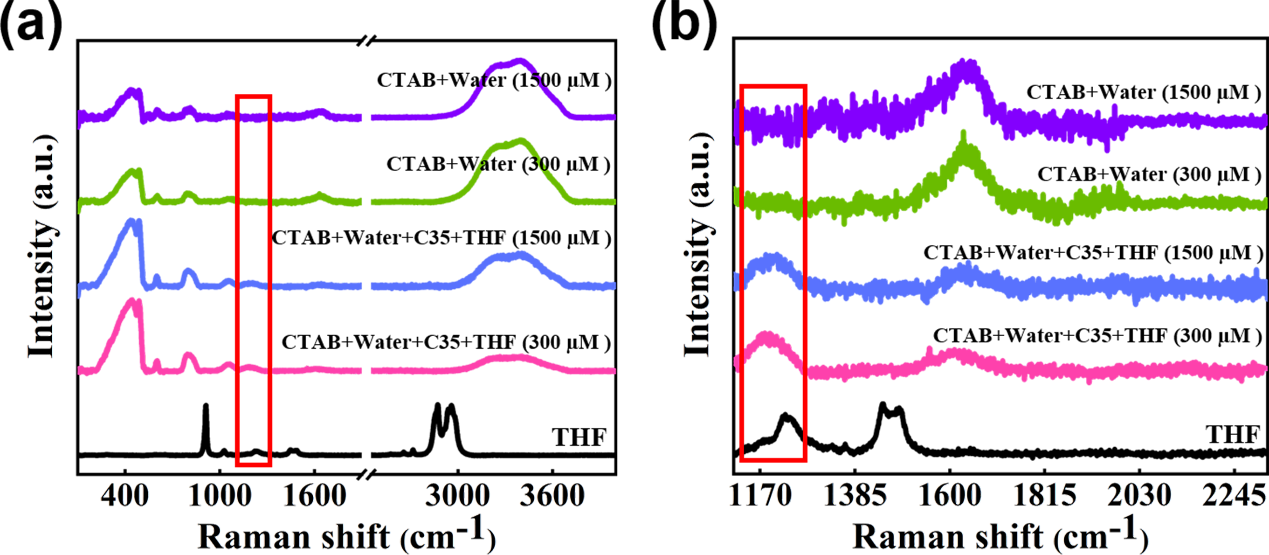
**

**Figure S3.** (a) Raman spectra of pure THF, 300 µM and 1500 µM CTAB with C35 in THF. Also, the Raman spectra of individual CTAB aqueous solutions are compared. (b) Enlarged Raman spectra from 1110 cm^-1^ to 2320 cm^-1^.

To determine the presence of residual THF in surfactant solutions, the Raman spectra of representative 300 µM and 1500 µM CTAB surfactants with C35 probe in THF are measured. In this case, the trace C35 stock solution with THF was likewise evaporated overnight on a shaker at room temperature. For comparison, the Raman spectra of pure THF, isolated 300 µM and 1500 µM CTAB aqueous solutions are also shown. Figure S3a shows the Raman spectra of five samples from 100 cm^-1^ to 4000 cm^-1^. The enlarged view of the red region in Figure S3a is demonstrated by Figure S3b. In Figure S3b, the Raman peak of the surfactant solutions with the C35 in THF and the pure THF exhibits a clear overlap at 1224.21 cm^-1^. It coincides with the Raman peak of THF at 1218.28 cm^-1^ obtained from theoretical simulations, attributed to an asymmetric rocking vibration of H-C-H. The individual surfactant aqueous solutions are absent from the Raman shift at 1224.21 cm^-1^. Hence, the comparison of Raman spectra indicates that the hydrophobic C35 was solvated by remaining trace THF and incorporated into the CTAB surfactant solubilized by water.


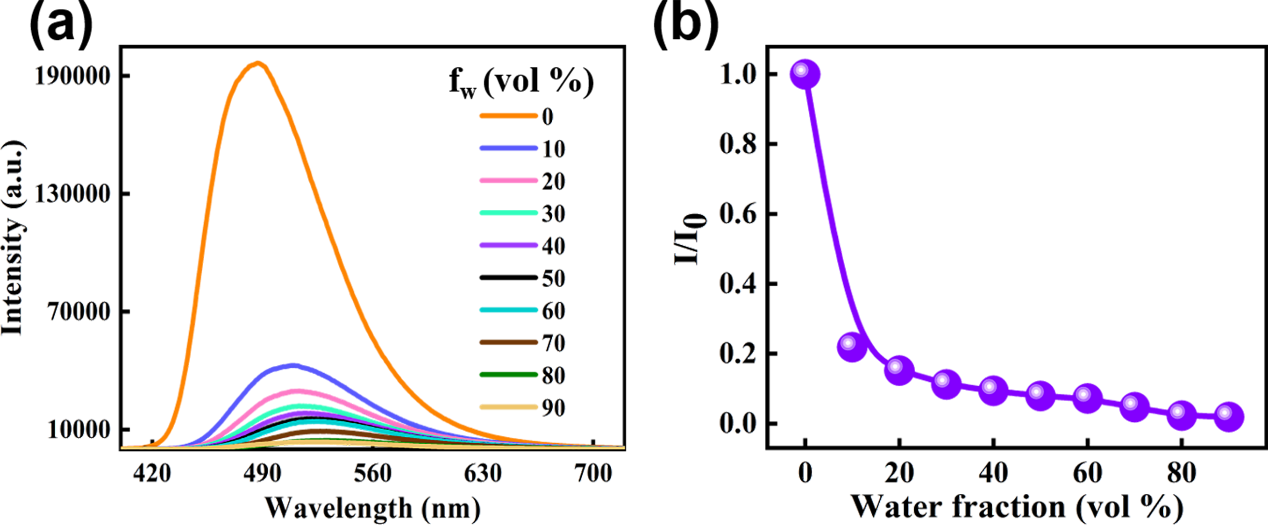


**Figure S4.** a) Fluorescence spectra of C35 in THF/water mixtures with different water fractions (f_w_). b) Plot of I/I_0_ values as a function of f_w_, where I_0_ represents the fluorescence intensity of C35 in pure THF.


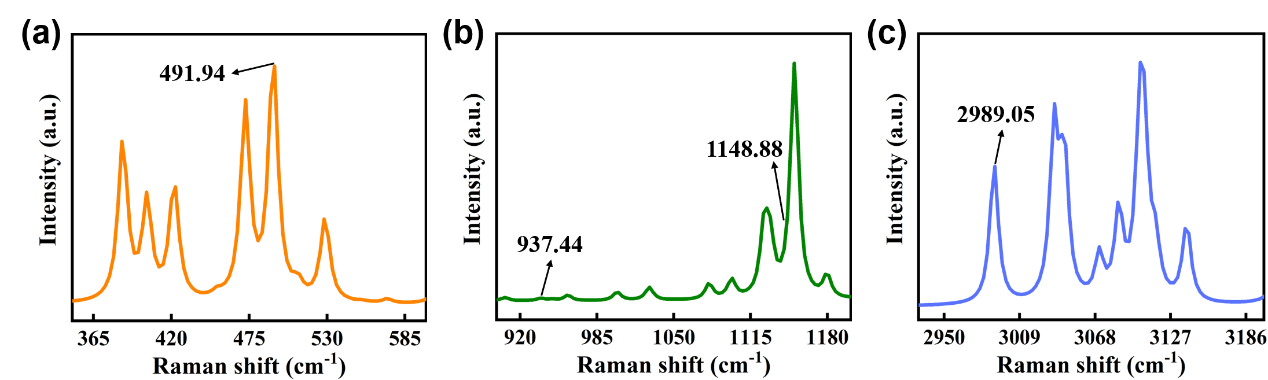


**Figure S5.** Calculated Raman spectra: a) 350-600 cm^-1^. b) 900-1200 cm^-1^. c) 2930-3200 cm^-1^.

**Table S1.** Attributed Raman shifts (cm^-1^) in the experiment with the aid of theoretical simulations.

| Experimental Frequency | Theoretical Frequency | Assignment |
| --- | --- | --- |
| 492.26  912.36  1148.11  2959.85 | 491.94  937.44  1148.88  2989.05 | C-N bend  C-H rock and C-N stretch  C-H bend  C-H stretch |

References

[1] B. Lin, H. Su, R. Jin, D. Li, C. Wu, X. Jiang, C. Xia, Q. Gong, B. Song, H. Ai, *Sci. Bull.* **2015**, *60*, 1272.

[2] J. Ren, X. Ren, Y. Li, J. Liu, S. Yuan, G. Wang, *Food Chem.* **2023**, *427*, 136707.

[3] G. Riess, *Prog. Polym. Sci.* **2003**, *28*, 1107.

[4] T. J. V. Prazeres, M. Beija, F. V. Fernandes, P. G. A. Marcelino, J. P. S. Farinha, J. M. G. Martinho, *Inorg. Chim. Acta* **2012**, *381*, 181.

[5] A. Jayaraman, *Rev. Mod. Phys*. **1983**, *55*, 65.

[6] Y. Wang, M. Bykov, I. Chepkasov, A. Samtsevich, E. Bykova, X. Zhang, S.-q. Jiang, E. Greenberg, S. Chariton, V. B. Prakapenka, A. R. Oganov, A. F. Goncharov, *Nat. Chem.* **2022**, *14*, 794.

[7] G. J. Piermarini, S. Block, J. D. Barnett, R. A. Forman, *J. Appl. Phys.* **1975**, *46*, 2774.

[8] C. Ruckebusch, M. Sliwa, P. Pernot, A. de Juan, R. Tauler, *J. Photoch. Photobio. C* **2012**, *13*, 1.

[9] J. Yang, J. Jing, W. Li, Y. Zhu, *Adv. Sci.* **2022**, *9*, 2201134.

[10] M. J. Frisch, G. W. Trucks, H. B. Schlegel, G. E. Scuseria, M. A. Robb, J. R. Cheeseman, G. Scalmani, V. Barone, B. Mennucci, G. A. Petersson, H. Nakatsuji, M. Caricato, X. Li, H. P. Hratchian, A. F. Izmaylov, J. Bloino, G. Zheng, J. L. Sonnenberg, M. Hada, M. Ehara, K. Toyota, R. Fukuda, J. Hasegawa, M. Ishida, T. Nakajima, Y. Honda, O. Kitao, H. Nakai, T. Vreven, J. A. Montgomery Jr., J. E. Peralta, F. Ogliaro, M. Bearpark, J. J. Heyd, E. Brothers, K. N. Kudin, V. N. Staroverov, T. Keith, R. Kobayashi, J. Normand, K. Raghavachari, A. Rendell, J. C. Burant, S. S. Iyengar, J. Tomasi, M. Cossi, N. Rega, J. M. Millam, M. Klene, J. E. Knox, J. B. Cross, V. Bakken, C. Adamo, J. Jaramillo, R. Gomperts, R. E. Stratmann, O. Yazyev, A. J. Austin, R. Cammi, C. Pomelli, J. W. Ochterski, R. L. Martin, K. Morokuma, V. G. Zakrzewski, G. A. Voth, P. Salvador, J. J. Dannenberg, S. Dapprich, A. D. Daniels, O. Farkas, J. B. Foresman, J. V. Ortiz, J. Cioslowski, D. J. Fox, *Gaussian 16*, revision B 01, Gaussian, Inc, Wallingford, CT, **2016**.

[11] A. D. Becke, *Phys. Rev. A* **1988**, *38*, 3098.

[12] L. X. Zhu, Q. Zhou, B. F. Cao, B. Li, Z. R. Wang, X. L. Zhang, H. Yin, Y. Shi, *J. Mol. Liq.* **2022**, *347*, 118365.

[13] T. Lu, F. Chen, *J. Comput. Chem.* **2012**, *33*, 580.

[14] E. Cancès, B. Mennucci, J. Tomasi, *J. Chem. Phys.* **1997**, *107*, 3032.
